# Temporal and scalar variations affect resource use of northern bobwhite broods

**DOI:** 10.1002/ece3.8161

**Published:** 2021-09-30

**Authors:** Bradley W. Kubečka, Theron M. Terhune, James A. Martin

**Affiliations:** ^1^ Warnell School of Forestry and Natural Resources University of Georgia Athens Georgia USA; ^2^ Tall Timbers Research Station and Land Conservancy Tallahassee Florida USA; ^3^ Warnell School of Forestry and Natural Resources Savannah River Ecology Lab University of Georgia Athens Georgia USA

**Keywords:** *Colinus virginianus*, prescribed fire, resource use, supplemental feeding, temperature, trade‐offs

## Abstract

Disparate resource use originating from phenology of biotic resources, abiotic conditions, and life cycles of exploiting organisms underscores the importance of research across time and space to guide management practices. Our goal was to evaluate resource use of northern bobwhite (*Colinus virginianus;* bobwhite) at two spatial scales and across three age classes, from hatching through a period of the postjuvenile molt. Our study was conducted at Tall Timbers Research Station, Tallahassee, FL, USA—situated in a landscape subjected to small scale (<20 ha) prescribed fires on a 2‐year fire rotation. We predicted prescribed fire, disking, and supplemental feeding would dictate resource use, but effects would depend on time since fire, brood age, and time of day. We predicted vegetation and temperature would govern roost use by broods, but these effects would also depend on age. We radio‐tracked 62 broods 21–35 times / week during May–October 2018 and 2019. Broods were less likely to use areas with large proportions of hardwood drains but favored sites with greater proportions of burned uplands, regardless of the time of day. Broods were less likely to use areas at greater distances from supplemental feed; this relationship had no interaction with age but was stronger later in the nesting season (>July 15). Broods were more likely to use areas with greater proportions of fallow fields during the day than for roosting. Broods used roosts with more woody cover and visual obscurity than at available sites. Roosts consisted of less grass and bare ground. However, these effects interacted with age; broods used sparser cover at older ages. Neonate broods were more likely to use cooler roosts with greater thermal stability, but this effect was reversed for juveniles. Broods may alter resource use with changes in vulnerabilities to threats such as thermal risks and predation.

## Graphical abstract

We documented resource use of a ground‐dwelling precocial bird in a fire‐managed landscape was conditional on multiple spatially and temporally variable factors. Particularly, our findings demonstrated that resource use was largely dependent on scale, physiological development, behavioral activity, and land management practices—some of which interacted with each other to create complex relationships.

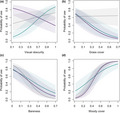

## 1 INTRODUCTION

Implications of differential resource use, manifesting through various spatial and temporal hierarchical processes, are a common and growing theme in ecology (Johnson, 1980; Kennedy & Gray, 1993; Strickland & McDonald, 2006). There is an increasing recognition that resource use occurs in flux with phenology of exploited biotic resources, physiological development of the exploiting species, abiotic conditions, and many other spatially and temporally variable factors (Rettie & Messier, 2000; Sinnott et al., 2021; Tsalyuk et al., 2019; Van der Merwe & Marshal, 2012). Studies without context of temporal and spatial scales may give rise to vastly opposing interpretations of resource use (Hernández, 2020; Hobbs, 2003). Accordingly, the processes of selection for a given species require research across multiple scales and regions to describe disparate behavior under varying conditions which in turn governs locally relevant management practices (Kauffman et al., 2009).

The northern bobwhite (*Colinus virginianus,* hereafter bobwhite) is a ground‐dwelling precocial game bird and one of the most studied avian species in the world (Cornell Lab of Ornithology, 2020; Scott, 1985). Studies of habitat and resource use are numerous (McGrath et al., 2017; Sands et al., 2012; Singh et al., 2011; West et al., 2012; Williams et al., 2000). Notably, however, bobwhite exhibit remarkable adaptive plasticity and occupy areas that range from semi‐arid shrublands to mesic forests to midwestern prairies (Guthery & Brennan, 2007). As such, though seemingly exhaustive, research on resource use has provided necessary information for management in vastly different regions. Most resource use studies have focused on adults, and much less information is available pertaining to resource use and ecology of young (Roseberry & Klimstra, 1984; Terhune et al., 2020). Additionally, prior studies have largely concentrated on diurnal activity (see Hiller & Guthery, 2005; Klimstra & Ziccardi, 1963; Taylor et al., 1999 for exceptions). Furthermore, bobwhite is a species of precipitous decline whereby survival of young is surmised to be highly influential to population dynamics (McConnell et al., 2018; Palmer et al., 2019; Sandercock et al., 2008; Sauer et al., 2017; Terhune et al., 2019; Yeiser et al., 2020). Therefore, if resource use by bobwhite young is different compared with nonbreeding resource use of adults, the time of day, or across different stages of physiological development, then management recommendations based on current information may not reflect the complexity of habitat requirements for bobwhite.

Habitat of bobwhite broods is conventionally described as vegetation communities of native forb, shrub, grass, and bare ground (Carver et al., 2001; Collins et al., 2009; Martin et al., 2009; Taylor & Burger, 2000; Taylor et al., 1999). Within the southeastern coastal range, the frequent application of prescribed fire is a common practice used to create and maintain a seral community resembling this vegetation composition (Brennan et al., 1998; Rother et al., 2020). Under this management approach, low‐intensity fires are typically applied at small scales (15–30 ha) biennially during February through May (Palmer & Sisson, 2017). While early spring fires are required to maintain appropriate seral communities for bobwhite habitat, vegetation used for concealment from predators is removed immediately prior to nesting season, occurring from April to September (Stoddard, 1931). Conversely, recently burned patches facilitate foraging efficiency by increasing the rate of mobility for bobwhite broods and the abundance of phytophagous arthropods, which comprise a large component of their diet (Burke et al., 2008; Hurst, 1972; Manley et al., 1994; Stoddard, 1931). Thus, considerable trade‐offs in the use of cover types may occur depending on time since burning, brood nutritional requirements, and ability to escape or evade predation.

In addition to prescribed fire, another popular management activity to promote favorable brooding conditions is nongrowing season (November–March) disking, which stimulates forb growth and thereby arthropod abundance. Reported use and importance of fallow fields is equivocal, however (Anderson et al., 2009; Carver et al., 2001; Palmer et al., 2012; Parsons et al., 2000). Properties intensively managed for bobwhite also commonly engage in supplemental feeding by broadcasting milo (*Sorghum bicolor*) into areas used by bobwhite (Sisson et al., 2000; Wellendorf, Palmer, & Sisson, 2017). The utility of this practice too has been argued as futile, especially for bobwhite young which forage primarily on arthropods at early ages (Doerr, 1989; Doerr & Silvy, 2002).

Collectively, inconsistent implications of resource use by broods may in fact arise from disconnects in scale, age, space, and/or time. That is, brood activity (e.g., foraging and roosting) or age might dictate the relative importance of use for various resources and conditions. Compared to foraging and diurnal habitat use, relatively little is documented about the use of roost sites by broods (see Hiller & Guthery, 2005; Taylor et al., 1999 for exceptions). Previous studies have suggested bobwhite use roost sites relatively sparse in vegetation as a mechanism for easily escaping predators if encountered (Klimstra & Ziccardi, 1963; Perkins et al., 2014). Alternatively, when the ability to evade predation is constrained by limited mobility (e.g., during nesting, brooding), prey may alter behavior by using sites that aid in concealment rather than facilitating escape (Lind et al., 2010; Wiebe & Martin, 1998). Moreover, in a pyric landscape, many of these factors may interact through time.

Abiotic conditions such as ground surface temperature may also be important factors in roost site use (Hiller & Guthery, 2005; Klimstra & Ziccardi, 1963). Notably, management such as prescribed fire that affects vegetation structure may also affect microclimate conditions and selection (Anthony et al., 2020; Carroll et al., 2017; Hovick et al., 2014). Particularly, sites devoid of vegetation after burning that are exposed to higher solar radiation may be warmer at dusk but should lose heat faster and become cooler by morning. Albeit precocial, bobwhite poorly thermoregulate until approximately 3–4 weeks of age and excessive heat, chilling, and wetting is of heightened concern for survival (Blem & Zara, 1980; Borchelt & Ringer, 1973; Spiers et al., 1985). Understanding the trade‐offs in resource use at various scales, ages, and periods in relation to management practices may refine the application of current land management practices across time and space.

Our goal was to evaluate diurnal and nocturnal resource use of bobwhite broods at two spatial scales (e.g., third and fourth orders; Johnson, 1980) and across three age classes (e.g., neonate, chick, and juvenile) in the southeastern coastal range. Our objectives were to first evaluate how the proximity to supplemental feed, landscape composition, and age affected space use by bobwhite broods within their home range (i.e., third order) (Johnson, 1980). Presuming burned uplands supported a greater abundance of arthropods for bobwhite young (Burke et al., 2008; Hurst, 1972), we predicted that the use of areas in closer proximity to supplemental feed would decrease with increasing proportions of surrounding burned upland habitat. Moreover, we predicted broods would use sites in closer proximity to supplemental feed with increasing age as diets become more inclusive. However, we hypothesized the use of burned uplands would depend on recovery time of the burned area. Thus, we predicted that the use of burned uplands would be greater later in the nesting season after burned patches had time to recover. Likewise, we predicted the use of areas near supplemental feed would also decrease later in the nesting season as natural foods became more abundant. At the third order, we further sought to evaluate the use of fallow fields (disked during January) and predicted that broods would favor fallow fields. We predicted that broods would be less likely to use landcover types such hardwood drains and hammocks because they do not support favorable vegetation communities. Lastly, we hypothesized that resource use would depend on whether broods were roosting or foraging. We predicted greater use of areas with higher proportions of burned uplands during the day because these patches are known to facilitate efficient foraging (Burke et al., 2008; Hurst, 1972). However, we expected broods would be less likely to use burned uplands for roosting because of sparser vegetation within these patches and potential negative influences of microclimate. We predicted broods would be equally unlikely to use drains for roosting as for diurnal activity and the use of fallow fields would be similar for roosting and diurnal activity.

At the point scale (1 m^2^; fourth order), we evaluated vegetation and temperature attributes at roost sites. Again, we hypothesized brood age would interact with effects of temperature and vegetation. We presumed hypothermia from cool and wet conditions was an imminent threat to young broods on our study site (Terhune et al., 2019) and thereby predicted broods would use warmer roost sites compared with available locations and that this effect would be stronger for younger broods. Similarly, we predicted broods would use sites with greater thermal stability and this effect would become less prominent for older broods. Lastly, we predicted broods would use roost sites with greater amounts of visual obscurity and bare ground as described in previous studies (Hiller & Guthery, 2005; Taylor et al., 1999).

## 2 MATERIALS AND METHODS

### 2.1 Study area

**FIGURE 1:**
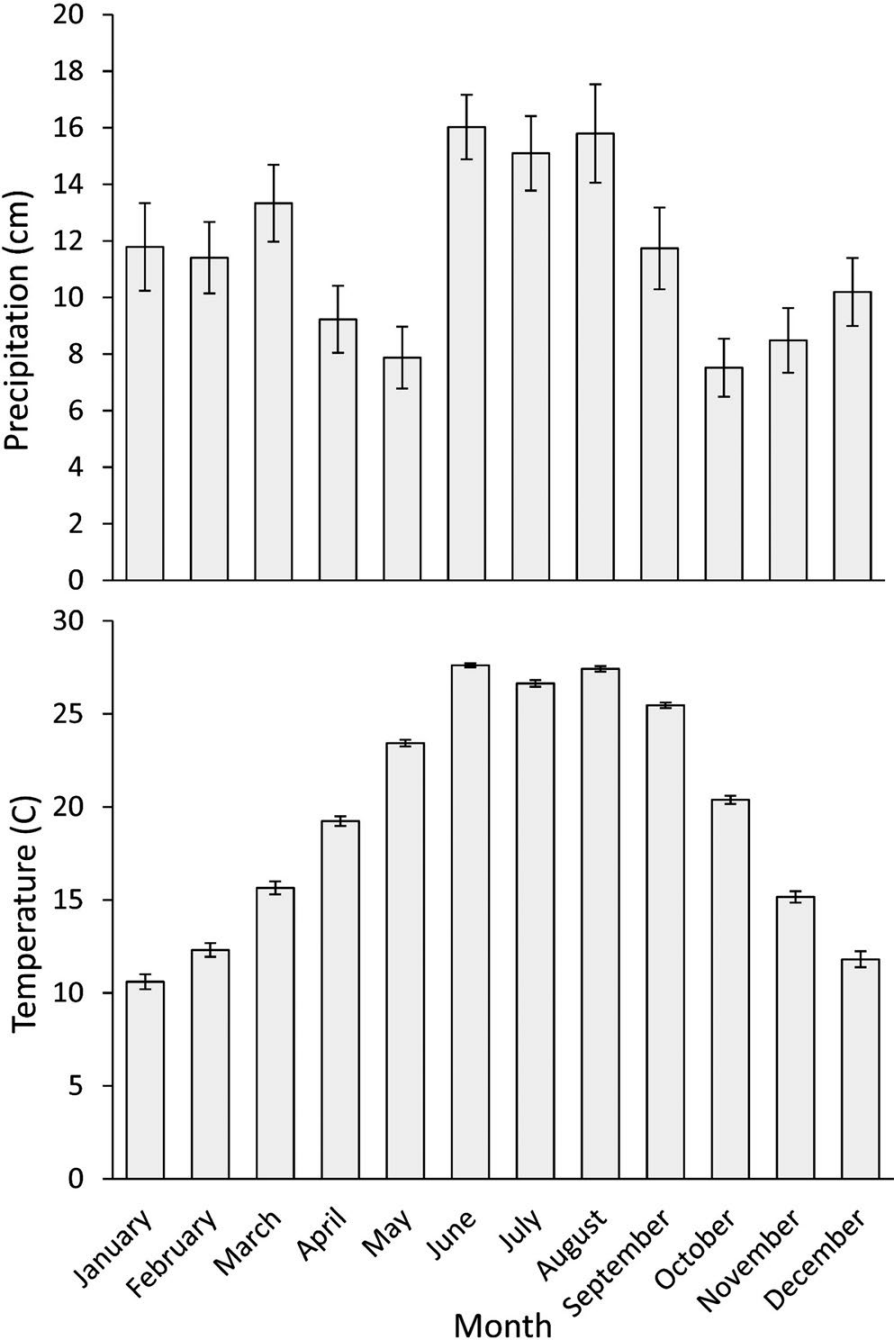
Mean monthly temperature and rainfall during 1988–2018 at Tall Timbers Research Station, Tallahassee, Florida, USA (PRISM Climate Group, Oregon State University 4‐km grid resolution). Error bars represent standard error of the mean

Our study was conducted on Tall Timbers in northern Leon County, Florida, USA, during 2018–2019. Tall Timbers is a 1,568‐ha area located in the Tallahassee Hills (colloquially known as the “Red Hills”), a southwestward extension of the Tifton Uplands physiographic province (Means, 2017). The Red Hills is characterized by rolling topography with sandy or loamy soils at depths less than 50 cm and clay below (Means, 2017). Tall Timbers is largely comprised of Faceville, Dothan, and Orangeburg sandy loam soil series (Natural Resources Conservation Service [NRCS], 2021). Elevation on Tall Timbers ranged from 40 to 70 m with high relief (10–20 m/0.1 km) (Means, 2017). The climate of Tall Timbers is warm temperate with a 30‐year (1988–2018) mean annual rainfall of 138.5 cm (PRISM Climate Group, 2020) and mean monthly temperatures ranging from 10.6°C in January to 27.6°C in June. The months from June to August, during peak hatching and brooding, are the wettest months of the year (Figure 1; PRISM Climate Group, 2020).

Tall Timbers is comprised of upland pine forests (66%), annually disked fields (13%), and hardwood drains (21%) (Terhune et al., 2019). Annually disked fields were disked during January each year and had an average area of 0.95 ha (range: 0.10–5.10 ha). Field vegetation composition was largely dominated by annual ragweed (*Ambrosia artemisiifolia*) and/or partridge pea (*Chamaecrista fasciculata*). Drains were dominated by arborescent live oak (*Quercus virginiana*), water oak (*Q. nigra*), laurel oak (*Q. laurifolia*), sweetgum (*Liquidambar styraflua*), pignut hickory (*Carya glabra*), and American beech (*Fagus grandifolia*). Shortleaf pine (*Pinus echinata*), loblolly pine (*P. taeda*), longleaf pine (*P. palustris*), and live oak comprised the majority of upland canopy, whereas undergrowth was comprised of comparable proportions of grass, forb, and shrub cover. Common grasses included broomsedge bluestem (*Andropogon virginicus*), silver plumegrass (*Saccharum alopecuroides*), eastern gamagrass (*Tripsacum dactyloides*), and intermittent bahiagrass (*Paspalum notatum*). Common forbs included annual ragweed, partridge pea, goldenrods (*Solidago* spp.), beggar's lice (*Desmodium* spp.), and showy rattlebox (*Crotalaria spectabilis*). Woody shrubs and vines consisted of American beautyberry (*Callicarpa americana*), mockernut hickory (*C*. *tomentosa*), winged sumac (*Rhus copallinum*), sweetgum, sand blackberry (*Rubus cuneifolius*), and greenbrier (*Smilax* spp.).

**FIGURE 2:**
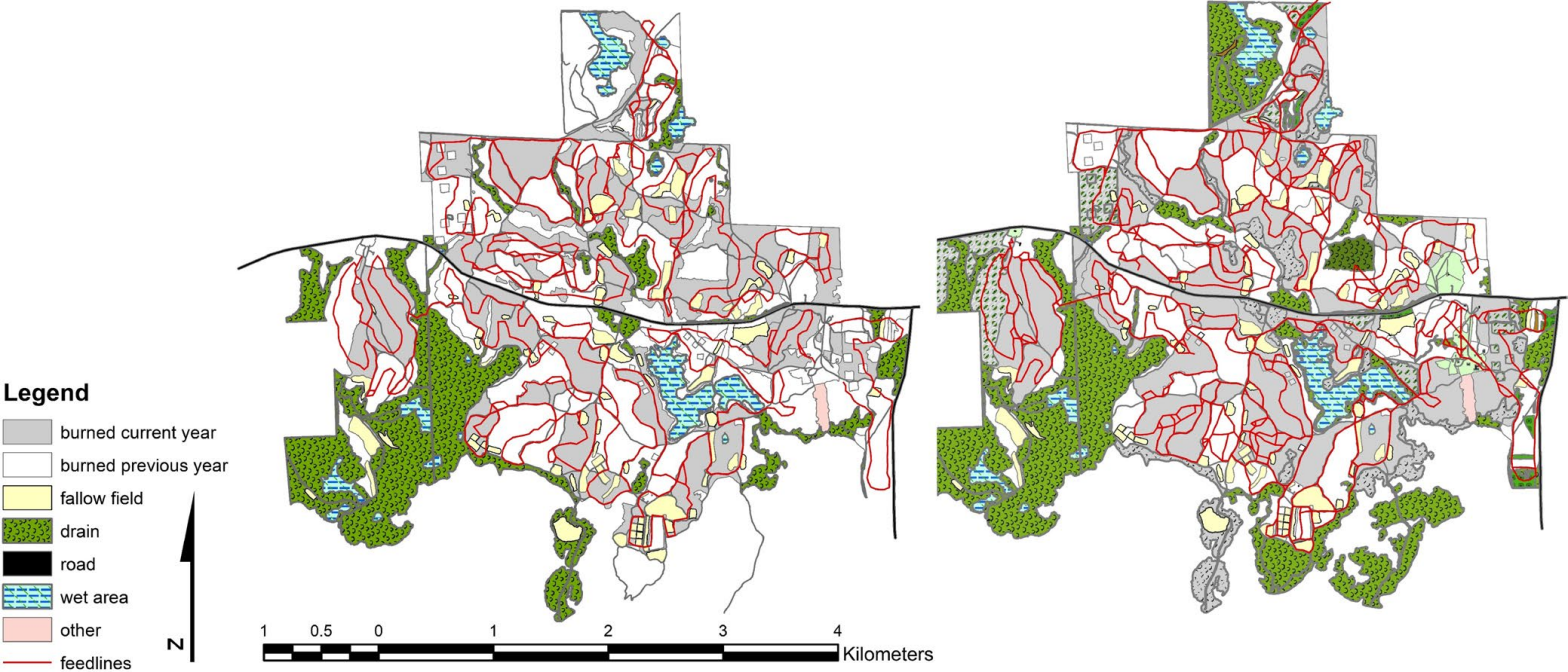
Landcover map of Tall Timbers, Leon County, FL, USA, during 2018 (left) and 2019 (right)

Uplands were subdivided by interior roads and subjected to prescribed fire during March–May on a 2‐year fire return interval (Figure 2). Thus, woody species listed previously that favor arborescent growth forms (e.g., sweetgum, mockernut hickory) were typically frutescent (a result of root crown regrowth) in the uplands of the study area. The practice of applying prescribed fire to the study site at this frequency has been conducted for multiple decades (Crawford & Brueckheimer, 2012) and largely limited the development of a midstory. Approximate size of cohesive burned and nonburned units was 17.8 ha (Figure 2; range: 4.4–47.2 ha). Supplemental feeding occurred by scattering approximately 3,300 kg of milo along designated feedlines on a biweekly schedule, year‐round. Feedlines were randomly placed throughout the area at approximately 1 km/14.8 ha (Figure 2). Placement of some feedlines changed from 2018 to 2019 due to varying equipment operators, but the application rate (kg) of milo spread throughout the habitat remained consistent during both years (Figure 2).

### 2.2 Field methods

#### 2.2.1 Radio telemetry

Adult bobwhites were trapped in walk‐in funnel traps during January, March, and November as part of a long‐term population monitoring study at Tall Timbers (Palmer et al., 2019; Palmer & Sisson, 2017; Terhune et al., 2019). Traps were placed across Tall Timbers in protective cover irrespective of road, feedline, or burn unit placement to maximize spatial coverage. A subsample of trapped individuals was marked with 6‐g necklace‐style VHF transmitters (American Wildlife Enterprises, Tallahassee, FL, USA) and monitored via radio telemetry at a rate of 3 times/week during October–April and daily during the nesting season (May–September). Locations were obtained via homing (White & Garrott, 1990) at an approximate distance of 30 m and recorded on a portable‐document format (PDF) map (Avenza Systems, Inc., Toronto, ON, Canada) using a GPS‐enabled mobile device (e.g., iPad, iPhone, or Android device). Maps were georeferenced with respective landcover types (e.g., burned upland, drain, and disked field). When individuals were documented in the same location after 2 consecutive visits, we assumed they were incubating. Incubating bobwhite were monitored daily until a nest fate was recorded (e.g., hatch, depredation). Upon a hatched nest, the incubating adult was tracked at a rate of 3 times per day. One location was obtained during the morning (08:00 hr–11:30 hr), one in the afternoon (13:00–17:00 hr), and one at night during roosting hours (~20:00 hr–07:00 hr). We began collecting roost locations approximately 45 min after official sunset to ensure broods had settled at their respective roosts for the night. We did not collect roost locations in the early morning hours (predawn) to avoid for the potential that a predator disturbed the roost during the night. Thus, our roost locations should represent sites initially selected by broods and unaffected by potential extraneous disturbances during the night that would have cause broods to move.

At 10–12 days of age, broods were captured as part of a concurrent study of chick survival using a corral method (Smith et al., 2003). All chicks that were captured were marked using patagial wing tags (National Band and Tag Co., Newport, Kentucky, USA) (Carver et al., 1999). If adults were captured without a brood, tracking ceased for that brood and tracking reverted to adult monitoring protocol. Data were retained up to the dates where observers could verify chick feces at roost disks if chicks were not present during brood captures. For broods captured with greater than 5 chicks, a subsample was marked with 0.75‐g VHF radio tags (American Wildlife Enterprises, Monticello, FL, USA), using a modified suture technique (Lunsford et al., 2019; Terhune et al., 2017, 2020). Telemetry data of marked chicks were used for multiple concurrent studies of chick survival which directed the minimum number of tagged chicks per brood. Chicks were tagged individually to ensure tracking persisted despite brood amalgamations, which are common among bobwhite (Brooks & Rollins, 2007; Faircloth, 2008; Faircloth et al., 2005). Thus, if chicks were adopted by another brood or orphaned by the brooding adult, we retained the ability to track chicks without the presence of a marked adult. Only chicks weighing greater than 15 g were marked to ensure radio‐packages were <5% of current body weight and had minimal influence on behavior and survival (Kenward, 2001). We continued to monitor broods with less than 5 chicks by tracking the incubating adult and using the presence of chick feces at roost disks as diagnostic sign to verify brood presence. Tracking ceased when chick feces was not present at the roost disk of the incubating adult, indicating brood mortality or amalgamation.

After chicks were individually tagged, tracking intensity increased to a rate of 5 locations per day during the week and 3 times per day on the weekends. The tracking schedule during the week consisted of 5 time slots: early morning (07:00–10:00 hr), mid‐morning (10:00–12:00 hr), early afternoon (12:00–15:00 hr), late afternoon (15:00–20:00 hr), and roost (20:00–07:00 hr). During weekend checks, the time slots included a morning (07:00–12:00 hr), afternoon (12:00–20:00 hr), and roost location (~20:00–07:00 hr). Daily tracking of broods continued until all chicks within a brood died or until 42 days of age.

#### 2.2.2 Temperature sampling

We sampled ground surface temperature at roost sites at predefined age intervals. At 3, 7, 14, 21, 28, 35, and 42 days of age, we marked roosts with flagging tape in each cardinal direction so they could be found the following morning. The morning (07:30 hr) following locating roosts, observers returned to the marked location and located the roost disk (identified by fecal dropping) within the flagged area. To evaluate the relative differences in temperature at roost sites to paired available locations, we deployed a DS1921G‐F50 Thermochron^®^ iButton^®^ (Maxim Integrated Products, Inc., Sunnyvale, CA) temperature sensor within the center of the roost disk and at a site 15 m in a random azimuth (determined by spinning a pencil). At 15 m from the roost site, the observer tossed the pencil in the air above and the point of its landing served as the precise random location. This distance was arbitrary but chosen to avoid spurious effects of spatial autocorrelation at close distances yet ensure the paired location was at a distance that was available to a brood. Sensors were glued to a 5‐cm, 11‐gauge roofing nail which secured its position flush with the ground surrounding it. Sensors were calibrated to collect a temperature reading every 20 min from 21:00 hr to 06:00 hr (28 readings). The sensor specifications allowed temperature readings ranging from −40°C to 85°C at 0.5°C increments. Accuracy of temperature readings within this range is 1°C (Maxim Integrated Products, Inc., Sunnyvale, CA).

Authors characterizing temperatures by bobwhite in the literature commonly report estimates of operative temperature by using temperature loggers fixed inside black, steel spheres (Carroll et al., 2015, 2016, 2018; Kline et al., 2019; Olsen et al., 2018). Operative temperatures represent the temperature experienced by an animal by accounting for solar radiation and convection. However, ambient temperature and operative temperature should be similar at night (i.e., given lack of solar radiation) assuming a constant wind speed (Guthery, 2002). Thus, we did not adjust temperatures recorded by the iButtons and assumed constant convection.

#### 2.2.3 Vegetation sampling

We collected measurements of vegetation during the same age intervals, and morning temperature sensors were deployed. At the roost site and paired random location also used for temperature sensors, we estimated visual obscurity (%) using a 1‐m^2^ cover board and vegetation composition (%) for 5 functional groups within a 1‐m^2^ quadrat frame. Functional groups which comprised vegetation composition included grasses, forbs, bare ground, litter, and woody/shrub cover. Species such as greenbrier and sand blackberry were categorized as woody/shrub cover, when present, due to their suffruticose or frutescent growth habits in the region. Burning biennially resulted in a sparse build‐up of litter that tended to be even at ground level. Therefore, we pooled the estimates of bare ground (i.e., exposed soil) and litter (e.g., pine needles) to an index called bareness. Pooled, they effectively represent traversability and lack of vertical vegetation structure.

Visual obscurity and vegetation composition were estimated using an image classification system. Images (3,024 × 4,032 pixel resolution) were classified using the point sampling software SamplePoint to standardize estimation among observers (Booth et al., 2006). Photographs were taken nadir (facing the quadrat, 1 m above) for estimates of vegetation composition. Photographs were taken 4 m south of the roost at a height of 1 m to estimate visual obscurity. The images were then cropped to the size of the cover board and quadrat and imported into SamplePoint. A systematic grid of 225 points was generated across the image and observers assigned a functional group to each point.

### 2.3 Analyses

#### 2.3.1 Third‐order use

Age of broods was a factor of particular interest in this study. Rather than use a continuous scale of age (in days) for our analyses, we developed age classes that coincided with physiologically relevant life stages (Table 1). We adopted this aging scheme so that our ages were linked to relevant life stages where we hypothesized resource use might interact (e.g., age class and proximity to supplemental feed).

**TABLE 1:** Life stages of northern bobwhite (*Colinus virginianus*) based on anatomical and physiological development

Age (days)	Life stage	Description
0–7	Neonate	Neonatal down predominates and is complete during this stage (Stoddard, 1931). Evaporative heat loss and heat production ratios vary significantly within the first 6 days (Spiers et al., 1985).
8–27	Chick	Pinfeather development begins at approximately 7 days of age and juvenal plumage development is well underway by 9 days (Stoddard, 1931). Short flights are achieved at approximately 13 days. The most substantial development in homeothermy occurs during the first 18 days of life (Spiers et al., 1985)
28–150	Juvenile	The postjuvenal primary molt begins at 28 days of age (Petrides & Nestler, 1943). The majority of feather growth occurs during this time, but the rate of homeothermic development is much slower (Spiers et al., 1985). Juveniles attain heat production rates comparable to adults at 65 days of age (Spiers et al., 1985).
>150	Subadult	The juvenile molt finishes at 150 days of age but is incomplete (9th and 10th primaries are retained). Sexual maturity is attained as early as 139 days of age under continuous lighting (Baldini et al., 1952). Average age to first egg laid is 174 days under these conditions. In a natural setting, bobwhite are sexually and somatically mature at this age but no evidence exists that suggest subadults copulate and lay within the first photo‐cycle.
First Nesting Season	Adult (First‐year breeder)	Individuals that survive to their first nesting season are sexually mature and considered adults. First‐year breeding adults retain “buffy tips” on primary coverts until their first postnuptial molt during August–October.

To evaluate resource use at the 3rd order, we first created binary raster images of landcover types from our general landcover map. Landcover types included burned upland, hardwood drains and hammocks, fallow fields, and nonburned uplands. We then used focal statistics in ArcMap 10.7 (ESRI, Redlands, CA, USA) to develop continuous rasters (1‐m^2^ grain) representing the proportion of each landcover type surrounding a cell within a 105‐m buffer. We defined the buffer size based on the average step length of broods in this study. As such, cell values for raster images represented the amount of area comprised by a given landcover type within a 3.46‐ha circular area surrounding a grid cell.

We generated 5 available points for each used location by creating circular buffers around used locations and then generating random locations within the buffers. This approach created choice sets among used locations amenable to discrete choice models. We chose a 1:5 use to availability ratio to ensure coefficient stability following an exploratory analysis of the data as suggested by Northrup et al. (2013). The scale at which availability of resources is defined can have major impacts on inference garnered from resource selection functions, and it is imperative that random locations are generated at a scale that matches selection processes (Boyce, 2006; Northrup et al., 2013). As such, the circular buffers that limited the extent of available locations were set to 1 standard deviation above the mean step length for each brood. We chose to vary distance of availability for each brood because some broods died soon after hatching whereas some broods survived the 42‐day monitoring period. We assumed broods soon after hatching did not have the same capacity for movement as broods at older ages and therefore would not have the same extent of available resources. The mean buffer size was 169 m (range: 3 m–266 m). The decision to constrain availability to 1 standard deviation above the mean step length was arbitrary. However, we intentionally avoided using the mean step length as a threshold on availability because this would inherently underestimate availability by restricting points to distances less than the mean step length. One instance occurred where a brood died the day of hatching, which is reflected by the lower continuum of buffer size.

We evaluated resource use at the 3rd order using Bayesian resource selection models with random effects (slopes and intercepts) for broods because we presumed observations within broods were not independent. The linear model included the effects of burned uplands (BU), hardwood drains (DR), fallow fields (FF), time of day (TOD), age class (Age), distance to supplemental feed (Feed), and interactions germane to our hypotheses. Nonburned uplands were highly correlated to the proportion of burned uplands (|r| = 0.82) and therefore excluded from the model. Correlation of burned uplands to hardwood drains (|r| = 0.02) and fallow fields (|r| = 0.24) were only weakly or moderately correlated and therefore retained. All variables were scaled, centered to the mean of the data before analyses for numerical convergence.

We specified normally distributed random intercepts with a mean of 0 and variance governed by a hyperparameter with a diffuse gamma distribution for broods. Priors on random slopes were specified using hyperparameters with diffuse normal and gamma distributions for the population mean and variance, respectively. We used the same shape and rate of gamma distribution (0.1, 0.1) for case and control sets (locid). We ran our model in the jagsUI package (Kellner, 2017) of program R (R Core Team, 2020) using the function “autojags.” We estimated the posterior distribution using Markov chain Monte Carlo (MCMC) sampling with 3 chains, a thinning rate of 3, and burn‐in of 500. The “autojags” function updated with iteration increments of 5,000 until our convergence criterion (Rhat <1.1) was met.

Our model was therefore:

$$\begin{array}{cc} \text{logit}(\theta_{i}) & =\beta_{0,s}+\beta_{\text{Age}}\times {\text{Age}}_{i}+\beta_{1,j}\times {\text{BU}}_{i}+\beta_{2,j}\times {\text{FF}}_{i}+\beta_{3,j}\times {\text{DR}}_{i}+\beta_{4,j}\times {\text{Feed}}_{i}+\beta_{5,j}\\ & \;\times {\text{TOD}}_{i}+\beta_{6,j}\times {\text{Season}}_{i}+\beta_{7,j}\times {\text{BU}}_{i}*{\text{TOD}}_{i}+\beta_{8,j}\times {\text{DR}}_{i}*{\text{TOD}}_{i}+\beta_{9,j}\\ & \;\times {\text{FF}}_{i}*{\text{TOD}}_{i}+\beta_{10,j}\times {\text{Feed}}_{i}*{\text{BU}}_{i}+\beta_{11,j}\times {\text{Feed}}_{i}*{\text{Age}}_{i}+\beta_{12,j}\\ & \;\times {\text{Feed}}_{i}*{\text{Season}}_{i}+\beta_{13,j}\times {\text{BU}}_{i}*{\text{Season}}_{i}+\varepsilon_{j} \end{array}$$

Priors were distributed as follows:

$$\beta_{0,s}∼\text{Normal}(0,\;\tau_{\text{locid}})$$

$$\beta_{\text{age}}∼\text{Normal}\left(0,\;0.001\right)$$

$$\beta_{k,j}∼\text{Normal}\left(\mu_{k},\;\tau_{k}\right)$$

$$\varepsilon_{j}∼\text{Normal}\left(0,\;\tau_{j}\right)$$

Whereas hyperparameters governing priors were as follows:

$$\tau_{k}∼\text{Gamma}\left(0.1,\;0.1\right)$$

$$\tau_{\text{age}}∼\text{Gamma}\left(0.1,\;0.1\right)$$

$$\mu_{k}∼\text{Normal}\left(0,\;0.001\right)$$

$$\tau_{\text{locid}}∼\text{Gamma}\left(0.1,\;0.1\right)$$

$$\tau_{j}∼\text{Gamma}\left(0.1,\;0.1\right)$$

The model likelihood followed a Bernoulli distribution where *Y_i_* ~ Bernoulli(ϴ*_i_*), and *Y* represents the use‐availability status for each location, *i*. Similarly, *j* represented each unique brood, *μ* represented population effects for *k* fixed effects, *β_age_* represented an additive effect of a categorical variable for age class, and locid coded for each choice set indexed by *s*.

#### 2.3.2 Fourth‐order use of roosts

**FIGURE 3:**
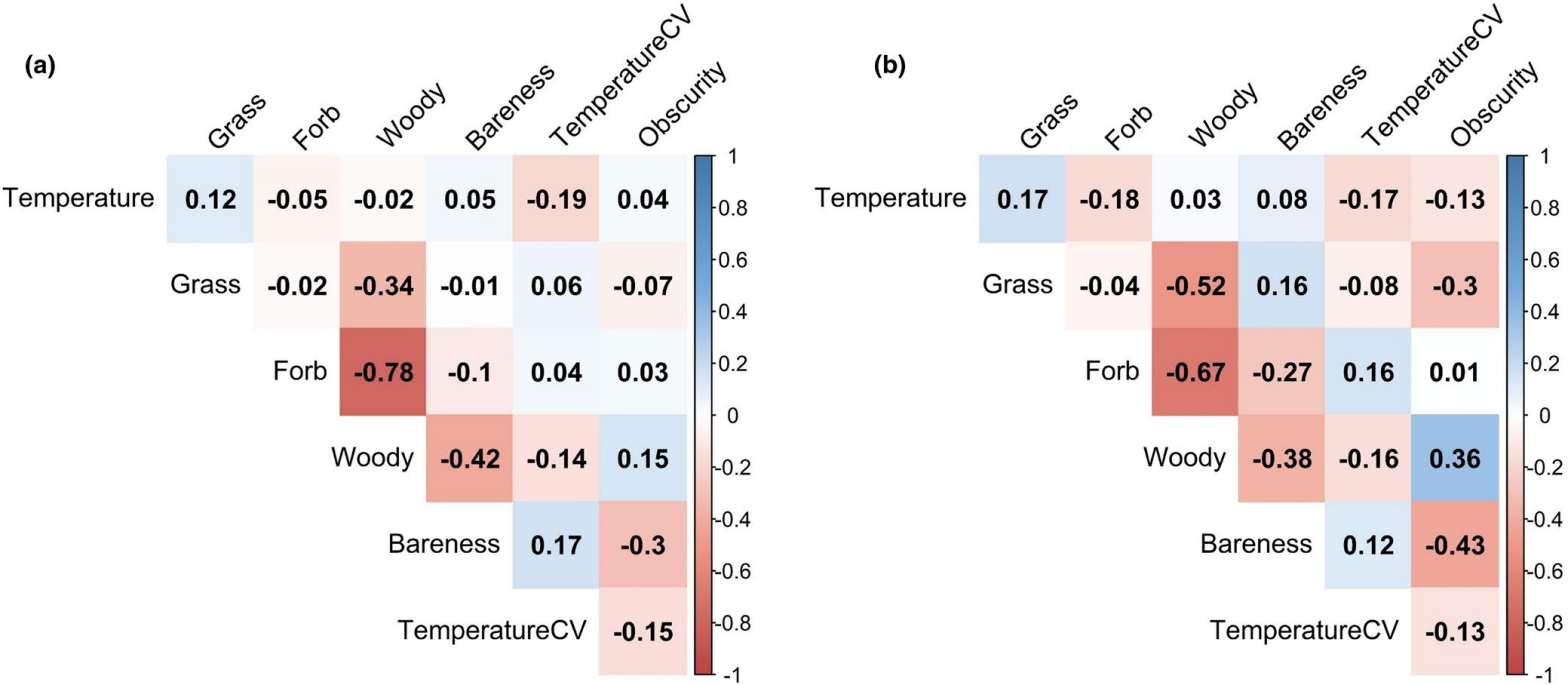
Correlograms of vegetation and temperature attributes at roost sites (a) and paired available locations (b) for northern bobwhite (*Colinus virginianus*) broods, Tall Timbers, Leon County, FL, USA. Temperature is average ground surface temperature from 21:00 to 06:00 hr whereas TemperatureCV represents the coefficient of variation in ground surface temperature during this same period. Labels are Pearson correlation coefficients

For this analysis, the choice set resembled a case–control design with a 1:1 use to availability ratio. Woody and forb cover were highly correlated (|r| = 0.72). Therefore, we removed forb cover as a predictor in the analysis because woody cover was germane to our hypotheses regarding postburn recovery. All other variables were moderate or weakly (<|r| = 0.42) correlated and therefore retained in the model (Figure 3).

Similar to the 3rd‐order analyses, we evaluated 4th‐order use with a Bayesian resource selection model, with random intercepts for broods. Each age class received its own intercept‐term ($\beta_{A}$), and each covariate was fit with a random‐slope of age $\left(\beta_{k,A}X_{k}\right)$, where *A* is each age group and *k* is each covariate. Each slope was drawn from a hierarchical random slope which allowed for more flexibility in how habitat selection varied as a function of age class. This also loosened the constraints of selection by one age class affecting selection of the previous and/or subsequent age class.

$$\text{logit}\left(\theta_{i}\right)=\beta_{A}+\sum_{k=1}^{K}\beta_{k,A}X_{k}+\varepsilon_{j}$$

Again, we specified priors for fixed effects with diffuse normal and gamma distributions for mean and precision, respectively. We ran our model in the jagsUI package (Kellner, 2017) of program R (R Core Team, 2020) using the function “autojags.” We estimated the posterior distribution using Markov chain Monte Carlo (MCMC) sampling with 3 chains, a thinning rate of 3, and 1,000 adaptations. The “autojags” function updated with iteration increments of 10,000 until our convergence criterion (Rhat < 1.1) was met.

## 3 RESULTS

We monitored 62 broods (*n*_2018_ = 34; *n*_2019_ = 28) during 25 May 2018–9 October 2018 and 25 May 2019–3 October 2019 and collected a total of 5,087 telemetry fixes (*n*_2018_ = 2,687; *n*_2019_ = 2,400). During 2018, 23 broods (68%) hatched in nonburned uplands, 10 broods (29%) hatched in burned uplands, and 1 brood (3%) hatched in a nonburned hardwood drain. In 2019, 21 broods (75%) hatched in nonburned uplands, 6 broods (21%) hatches in burned uplands, and 1 brood (4%) hatched in a fallow field. Telemetry fixes consisted of 1,626 roosts (*n*_2018_ = 931; *n*_2019_ = 695) and 3,461 diurnal locations (*n*_2018_ = 1,756; *n*_2019_ = 1,705). Mean number of fixes per brood was 79.0 ± 63.2 (*SD*; range: 2–166 fixes) during 2018 and 85.7 ± 54.9 (*SD*; range: 4–174 fixes) in 2019. The presence of a brood's incubating adult was verified using radio telemetry and occurred at 90.8% of all roosts (*n* = 1,476 roosts). The earliest age we documented incubating adults orphaning broods was 11 days of age; we ceased tracking broods when orphaning or adult mortality occurred before chicks were radio‐tagged which precluded tracking. This estimate of brood amalgamation is thereby conservative.

We collected temperature data at 176 paired locations comprised of 9,854 temperature observations. Paired locations were comprised of 70 neonate, 53 chick, and 53 juvenile brood roosts (Table 2). We collected vegetation measurements at 181 paired sites comprised of 74 neonate, 56 chick, and 51 juvenile brood roosts (Table 3). Discrepancies in vegetation and temperature sample sizes arose from malfunctions or mis‐calibration of iButtons, data corruption, and small mammal iButton kleptomania.

**TABLE 2:** Descriptive statistics of ground surface temperatures (°C) at roost sites and paired available locations of northern bobwhite (*Colinus virginianus*) broods, Tall Timbers, Leon County, FL, USA, during 2018–2019

	Mean (*SD*)			Range		
	Neonate	Chick	Juvenile	Neonate	Chick	Juvenile
Roost	23.8 (1.4)	24.6 (1.2)	24.3 (1.3)	19.5–28	21–28	19.5–28.5
Random	23.9 (1.5)	24.7 (1.3)	24.3 (1.3)	20–28	21.5–29	20.5–29

Note

Age classes are described as neonate (<8 days; *n* = 70 roosts), chick (8–27 days; *n* = 53 roosts), and juvenile (28–42 days; *n* = 53 roosts).

**TABLE 3:** Descriptive statistics of vegetation attributes (%) at roost sites (1 m^2^) and paired available locations of northern bobwhite (*Colinus virginianus*) broods, Tall Timbers, Leon County, FL, USA, during 2018–2019

		Mean (*SD*)			Range		
		Neonate	Chick	Juvenile	Neonate	Chick	Juvenile
Woody	Roost	50.7 (25.3)	52.7 (23.1)	50.6 (24.6)	2.7–90.7	0.9–91.6	3.6–97.8
	Random	33.9 (23.7)	41.4 (26.0)	44.0 (30.8)	0.0–93.3	1.8–92.9	0.0–98.7
Grass	Roost	6.4 (8.6)	5.8 (5.6)	8.7 (11.7)	0.0–62.2	0.0–24.4	0.0–66.2
	Random	11.2 (11.4)	8.5(8.4)	13.6 (16.5)	0.0–44.9	0.0–34.2	0.0–70.2
Forb	Roost	27.4 (20.0)	25.8 (22.3)	20.9 (19.1)	0.0–83.1	0.0–89.8	0.0–81.3
	Random	30.2 (20.5)	28.2 (24.0)	23.2 (23.0)	0.0–84.0	0.0–88.0	0.0–99.1
Bareness	Roost	15.5 (11.5)	15.8 (10.4)	19.7 (14.6)	0.9–59.6	0.4–45.7	0.4–74.7
	Random	24.7 (13.5)	21.9 (15.7)	19.2 (12.6)	2.7–74.7	1.3–64.0	0.0–49.8

Note

Age classes are described as neonate (<8 days; *n* = 74 roosts), chick (8–27 days; *n* = 56 roosts), and juvenile (28–42 days; *n* = 51 roosts). Bareness index describes the sum of bare ground and litter.

### 3.1 Third‐order use

Our model estimated broods were less likely to use areas with large proportions of surrounding hardwood drains but favored sites with greater proportions of burned uplands (Table 4). We observed probability of use for burned uplands and hardwood drains was similar for both roosting and diurnal activity. Counter to our prediction that probability of use for burned uplands would be lower early in the nesting season (due to short burn recovery time), the effect of burned upland was consistent throughout the nesting season (Table 4). Broods were more likely to use areas with greater proportions of fallow fields during the day and less likely for roosting (Table 4).

**TABLE 4:** Posterior means and credible intervals of third‐order habitat use of northern bobwhite (*Colinus virginianus*) broods, Tall Timbers, Leon County, FL, USA

Parameter	Mean	0.025	0.500	0.975	*f*
Burned upland	0.24	0.11	0.24	0.37	1.00
Hardwood drain	−0.17	−0.30	−0.16	−0.04	0.99
Fallow field	0.07	−0.05	0.07	0.19	0.89
Distance to feed	−0.10	−0.22	−0.10	0.02	0.95
Time of day	−0.01	−0.11	−0.01	0.08	0.60
Season	−0.56	−1.18	−0.53	−0.11	1.00
Burned upland*Time of day	−0.02	−0.11	−0.03	0.07	0.71
Hardwood drain*Time of day	−0.05	−0.18	−0.05	0.07	0.79
Fallow field*Time of day	−0.11	−0.22	−0.11	0.00	0.97
Distance to feed*Burned upland	0.09	0.01	0.09	0.16	0.99
Neonate [fixed]	0.00	0.00	0.00	0.00	1.00
Chick	−0.09	−0.18	−0.09	0.00	0.98
Juvenile	−0.02	−0.13	−0.02	0.09	0.63
Distance to feed*Neonate [fixed]	0.00	0.00	0.00	0.00	1.00
Distance to feed*Chick	−0.04	−0.16	−0.04	0.08	0.75
Distance to feed*Juvenile	0.06	−0.10	0.06	0.22	0.76
Burned upland*Season	0.05	−0.15	0.05	0.24	0.70
Distance to feed*Season	−0.16	−0.33	−0.16	0.02	0.97
tau.brood	0.40	0.25	0.39	0.61	1.00
tau.locid	139.04	99.03	136.97	189.58	1.00

Note

The *f* value describes the proportion of the posterior distribution with the same sign as the mean.

**FIGURE 4:**
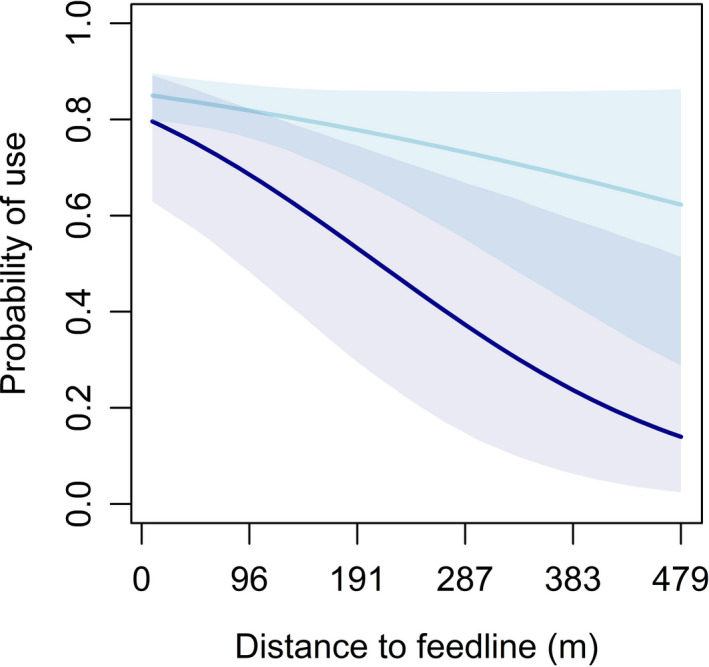
Marginal effects of distance to supplemental feed (m) on third‐order resource use by northern bobwhite (*Colinus virginianus*) broods during 2018–2019 at Tall Timbers, Leon County, FL, USA. Light blue represents early nesting season (≤July 15), and dark blue represents late nesting season (>July 15). Shading represents 95% Bayesian credible intervals

**FIGURE 5:**
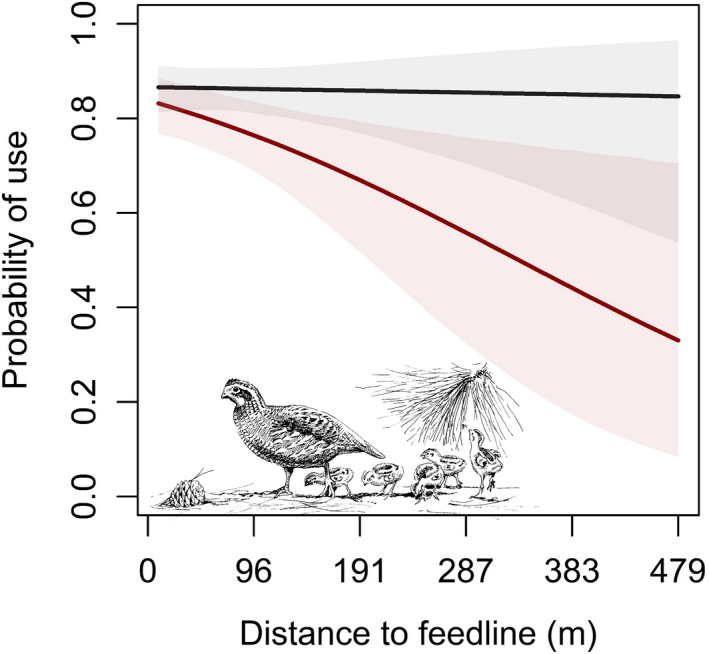
Marginal effects of distance to supplemental feed (m) on third‐order resource use by northern bobwhite (*Colinus virginianus*) broods during 2018–2019 at Tall Timbers, Leon County, FL, USA. Black represents a site where burned upland is held to 1 *SD* above the mean observed value of burned uplands, and dark red represents 1 *SD* below the mean observed value for burned uplands. Shading represents 95% Bayesian credible intervals

Broods were less likely to use areas at greater distances from supplemental feed, and counter to our prediction, this effect did not depend on age (Table 4). Also counter to our predictions, the effect of distance to feed was stronger later in the nesting season (>July 15) compared with early (≤July 15) (Table 4, Figure 4). The effect of supplemental feed tended to be less influential as the proportion of surrounding area with burned upland increased (Table 4, Figure 5). This supported our prediction that the effect of supplemental feed would be greater in nonburned uplands where arthropods were presumed to be in lower abundance and accessibility.

### 3.2 Fourth‐order use

**FIGURE 6:**
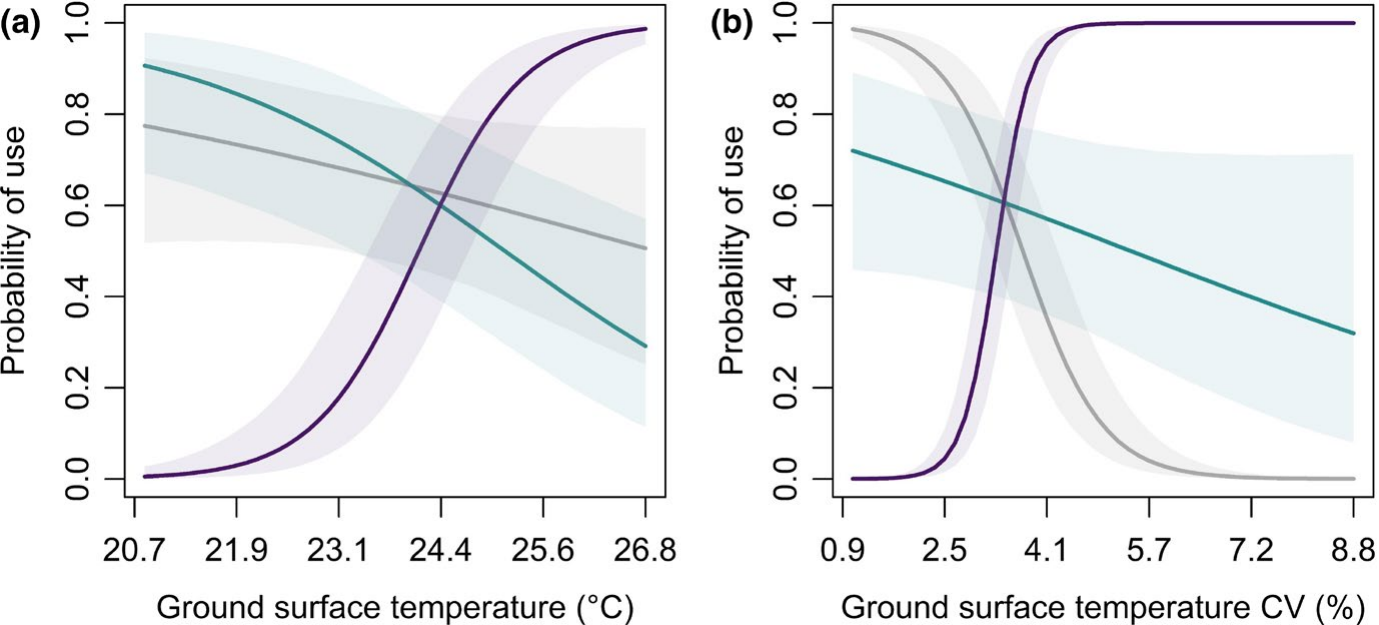
Marginal effects of mean ground surface temperature (a) and temperature stability (b) on roost site use across 3 ages classes of northern bobwhite (*Colinus virginianus*) broods during 2018–2019 at Tall Timbers, Leon County, FL, USA. Line colors code for age, where gray represents neonates (0–7 days), green represents chicks (8–27 days), and purple represents juveniles (28–42 days). X‐axes span for range of observed data. Shading represents 95% Bayesian credible intervals

**FIGURE 7:**
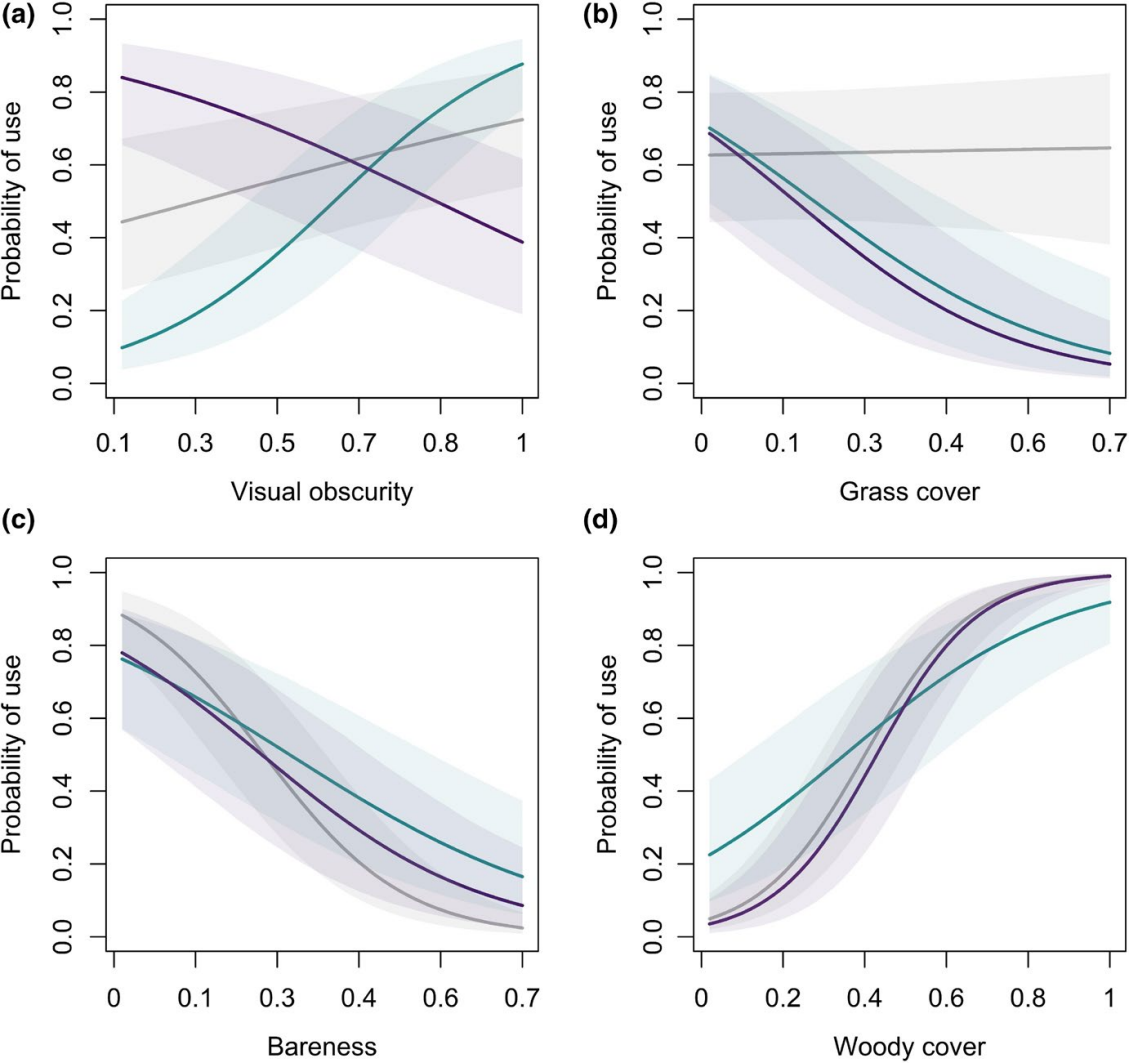
Marginal effects of vegetation attributes on roost site use across 3 age classes of northern bobwhite (*Colinus virginianus*) broods during 2018–2019 at Tall Timbers, Leon County, FL, USA. Line colors code for age, where gray represents neonates (0–7 days), green represents chicks (8–27 days), and purple represents juveniles (28–42 days). X‐axes span for range of observed data. Shading represents 95% Bayesian credible intervals

At the point scale, age interacted with effects of visual obscurity, grass cover, mean ground surface temperature, temperature stability, and the bareness index (Table 5; Figures 6 and 7). Counter to our predictions, neonates used roosts with cooler ground surface temperatures than paired available locations, and juveniles used roosts with comparatively warmer mean surface temperatures (Figure 6a). Interestingly, neonate broods used roosts with greater thermal stability (i.e., low coefficient of variation), but the effect was reversed for juveniles (Figure 6b). Overall, neonate broods used roosts with greater amounts of concealment and cover, whereas juvenile broods tended to use roost sites with less vegetation (Figure 7).

**TABLE 5:** Posterior means and credible intervals of fourth‐order, roost site selection of northern bobwhite (*Colinus virginianus*) broods, Tall Timbers, Leon County, FL, USA

Parameter	Mean	0.025	0.500	0.975	f
Neonate	−0.05	−0.62	−0.02	0.45	0.56
Chick	−0.14	−0.87	−0.07	0.37	0.66
Juvenile	−0.26	−1.14	−0.16	0.26	0.76
Temp*Neonate	−0.21	−0.53	−0.21	0.09	0.91
Temp*Chick	−0.56	−0.93	−0.56	−0.19	1.00
Temp*Juvenile	1.69	1.27	1.69	2.13	1.00
Woody*Neonate	2.04	1.77	2.04	2.33	1.00
Woody*Chick	0.98	0.71	0.98	1.26	1.00
Woody*Juvenile	2.12	1.73	2.12	2.52	1.00
Grass*Neonate	0.01	−0.13	0.01	0.16	0.57
Grass*Chick	−0.52	−0.75	−0.51	−0.29	1.00
Grass*Juvenile	−0.58	−0.76	−0.58	−0.40	1.00
TempCV*Neonate	−2.10	−2.43	−2.10	−1.78	1.00
TempCV*Chick	−0.28	−0.65	−0.28	0.07	0.94
TempCV*Juvenile	4.97	4.37	4.97	5.55	1.00
Bareness*Neonate	−1.04	−1.20	−1.04	−0.88	1.00
Bareness*Chick	−0.51	−0.67	−0.51	−0.34	1.00
Bareness*Juvenile	−0.66	−0.88	−0.66	−0.44	1.00
Obscurity*Neonate	0.31	0.13	0.31	0.49	1.00
Obscurity*Chick	1.09	0.90	1.08	1.28	1.00
Obscurity*Juvenile	−0.55	−0.72	−0.55	−0.38	1.00
Season	0.49	−0.03	0.48	1.06	0.97
Woody*Season*Neonate	−1.17	−1.50	−1.17	−0.86	1.00
Woody*Season*Chick	−0.69	−1.01	−0.69	−0.37	1.00
Woody*Season*Juvenile	−1.54	−1.91	−1.54	−1.17	1.00
tau.pair	0.14	0.10	0.13	0.18	1.00
tau.brood	30.39	0.61	3.44	215.22	1.00

Note

The *f* value describes the proportion of the posterior distribution with the same sign as the mean.

**FIGURE 8:**
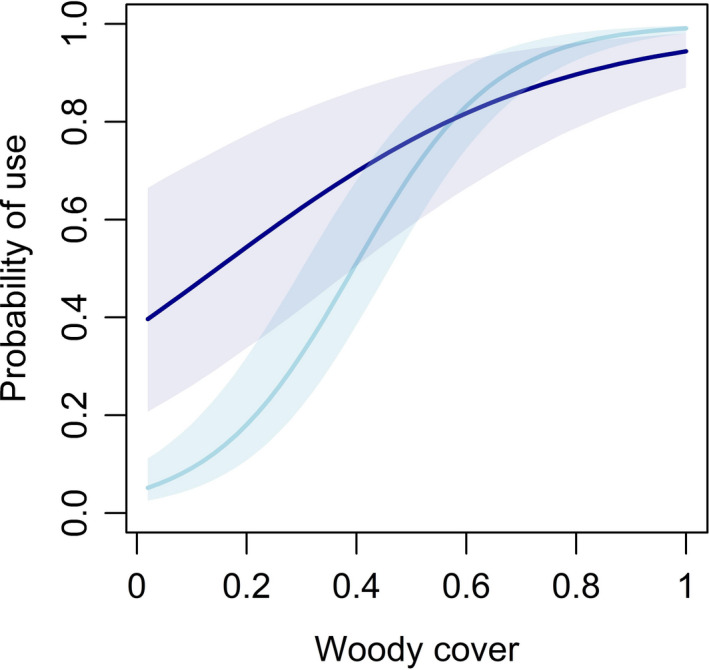
Marginal effects of woody cover on roost site use by neonate (0–7 days of age) northern bobwhite (*Colinus virginianus*) broods during 2018–2019 at Tall Timbers, Leon County, FL, USA. Light blue represents early nesting season (≤July 15), and dark blue represents late nesting season (>July 15). Shading represents 95% Bayesian credible intervals

Unlike other vegetation attributes, the effect of woody cover was not substantially influenced by age (Table 5)—broods of all ages were more likely to use roosts with greater amounts of woody cover (Figure 7d). Furthermore, our model estimated the effect of woody cover was stronger in the early nesting season compared with late nesting season (Table 5). The interaction of woody cover and time of nesting season, but not age, suggest woody cover provides important roosting cover for broods early in the nesting season when postburn recovery time is short, and regrowth of herbaceous cover limited (Figure 8).

## 4 DISCUSSION

We documented resource use of a ground‐dwelling precocial bird in a pyric landscape was conditional on multiple spatially and temporally variable factors. Particularly, our findings demonstrated that resource use was largely dependent on scale, physiological development, behavioral activity, and land management practices—some of which interacted with each other to create complex relationships. Bobwhite broods demonstrated dynamic resource use in this study, underscoring the importance of similar studies across the bobwhite geographic distribution where management, resources, and conditions differ. Properties managed similar to our study area support some of the highest density bobwhite populations in the United States (Palmer & Sisson, 2017). However, properties not purposefully managed for bobwhite within the same region may be completely unoccupied. Therefore, replicated studies even within the same region under different conditions (e.g., fire regime, food provisioning) are warranted.

Our results both supported and refuted the conclusions of Wellendorf, Palmer, and Bostick (2017) who found the effect of supplemental feed on space use for bobwhite in north Florida was greater early in the nesting season (defined as <June 30 by Wellendorf et al.) compared with late, whereby we observed the strength of distance to supplemental feed was stronger later in the nesting season (>July 15). Our study differed from Wellendorf, Palmer, and Bostick (2017) by exclusively focusing on broods, with or without adults whereas their emphasis was on breeding season resource use of adults, with or without broods. Furthermore, Wellendorf, Palmer, and Bostick (2017) began studying adult breeding season space use in April, whereas broods in this study were not available until late May each year. This timing, in relation to prescribed fire and covey breakup, could help explain more of the discrepancy (S. Wellendorf, Tall Timbers, personal communication).

We found that the effects of proximity to supplemental feed on resource use did not depend on age, suggesting that bobwhite broods may use grain sorghum more than suspected at early ages and when alternative foods are in lower availability, such as in nonburned uplands. Alternatively, it is possible that broods used feedlines for increased mobility, where vegetation was trampled from the act of distributing feed. Another hypothesis explaining this observation is that adults led broods to feedlines for their own benefit. Research on diet composition of bobwhite broods in tandem with resource use may serve to verify whether broods use feedlines as a food source or for other means.

The effect of fallow fields on resource use was not as strong as predicted and supports the conclusions of some (Carver et al., 2001; Hammond, 2001; Palmer et al., 2012), but not others (Crouch, 2010; McGrath et al., 2017; Parsons et al., 2000). Palmer et al. (2012) surmised these differences could be due to variation in soil type that would affect vegetation response to different management practices and abiotic conditions. Our results support these conclusions, given our study area was situated in a highly fertile clay loam which recovered quickly after prescribed fire and received ample precipitation. Moreover, we found that the effect of fallow fields on resource use depended on time of day, where broods were less likely to use field roosting. At the point scale, we found that broods favored sites with greater amounts of woody cover. It is likely that the use of fields for roosting was lower compared with diurnal activity because woody cover was limited in fields due to repeated annual disking.

The use of burned uplands by broods did not depend on early or late nesting season dates, which served as a proxy for postburn recovery time. In both 2018 and 2019, our sample of broods entered on 25 May, and the shortest burn recovery time for areas used by broods was 40 days. Hatches occurred throughout nesting season (May–September). Thus, age and time of nesting season should not have been confounded. Fertile soils and ample precipitation likely facilitated prompt vegetation recovery on our study site, but this effect may not be consistent for areas with different soils or in lower precipitation zones. The effect of hardwood drains on resource use of broods coincided with our predictions. Land managers have little control over topographical attributes and soil drainage properties; however, frequent burning of these areas may provide some utility during drought (Palmer et al., 2012).

We expected chilling and wetting would present an imminent threat to broods and therefore predicted trade‐offs in roost use based on vegetation and temperature. Neonates used roost sites that were slightly cooler than available locations, but the difference between mean temperatures at roosts and available locations was small (Table 2). Mean temperatures at roost sites for chicks and juveniles were similar to available locations (Table 2). Available locations were situated 15 m from roosts. Thus, the difference in temperature at this scale would not be expected to be large given Tobler's Law. We reconcile similar mean temperatures to differential probability of use partly due to the thermal stability documented at roosts sites. We documented roosts used by neonate broods were more thermally stable than paired available locations, suggesting vegetation at roosts likely moderated solar radiation during the day and heat loss throughout the night. A post hoc correlogram of temperature attributes to vegetation supports this hypothesis (Figure 3). As broods aged, the use of roost sites became characterized by sparser vegetation which could have led to less stable microclimates. Our correlogram supports this hypothesis, where coefficient of variation in temperature increased with increasing site bareness and decreased with increasing woody cover (Figure 3). Similar relationships of time since fire, vegetation, and microclimate have been noted elsewhere and were determined to affect space use of other ground‐dwelling birds (Anthony et al., 2020; Carroll et al., 2017).

**FIGURE 9:**
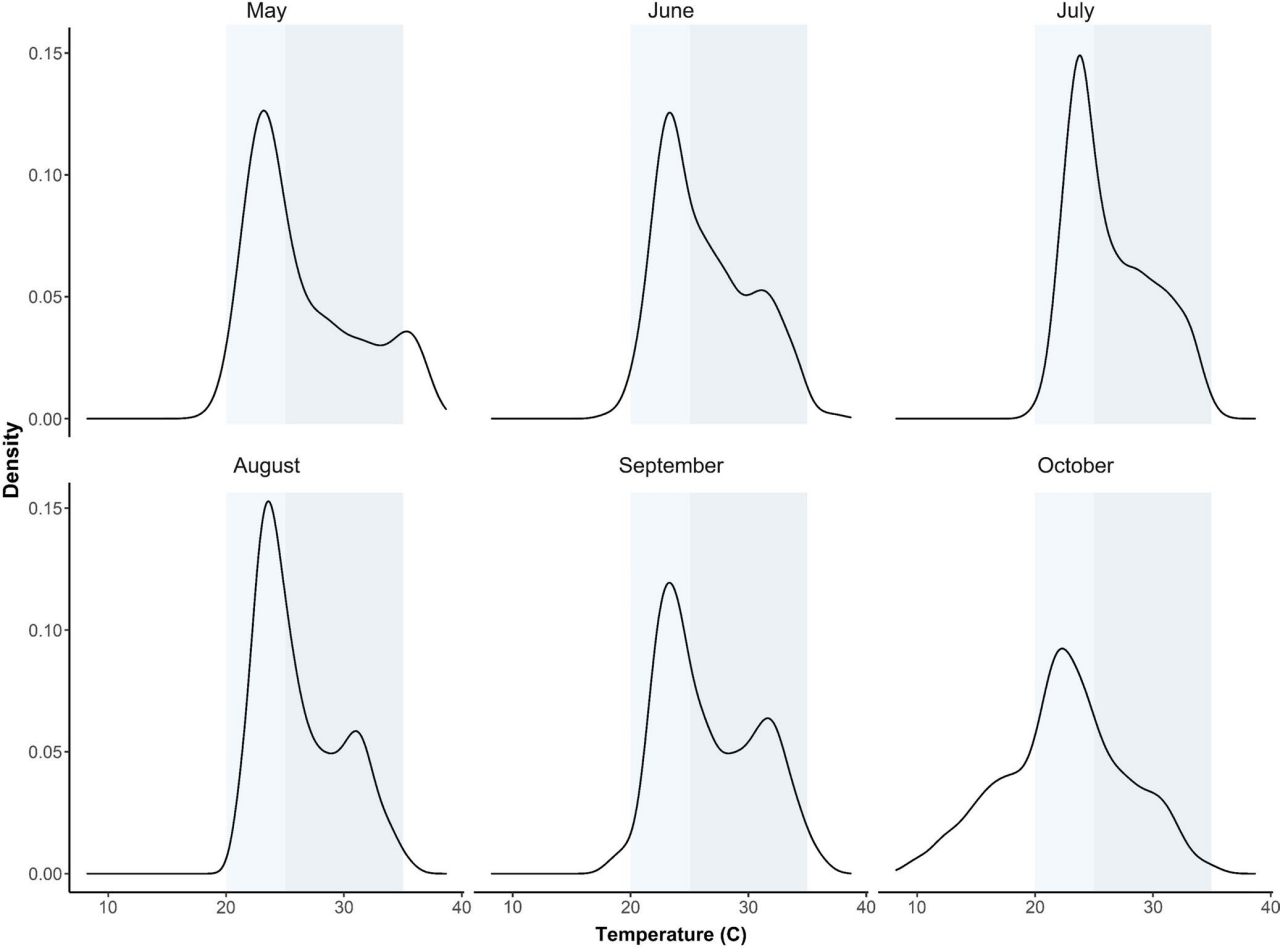
Density plots of ambient temperatures observed at Tall Timbers, Leon County, FL, USA, during northern bobwhite (*Colinus virginianus*) breeding season, 2018 and 2019. The weather station (Campbell Scientific, Logan, UT, USA) recorded temperature every minute at 1.5 m above ground level. Light blue shading denotes 20–35°C and dark blue 25–35°C, representing varying proposed ranges of thermoneutrality for northern bobwhite

Spiers et al. (1985) estimated a lower critical temperature threshold for bobwhite at 20°C, where individual bobwhite less than 14 days of age became immobile. By 18 days of age, bobwhite chicks are able to maintain stable body temperatures at ambient temperatures of 20°C (Spiers et al., 1985). The presence of a brooding adult and/or other individuals in a brood likely ameliorates the effect of ambient temperatures below this threshold. We observed ground surface temperatures at roosts falling below 20°C for broods less than 18 days of age on one occasion in our study; these temperatures were sustained from 04:00 to 06:00 hr, and parental care was documented. Given the range of ground surface temperatures we observed at roost sites (19.5°C–28.5°C) largely overlapped with the thermoneutral zone (Figure 9) and presence of other individuals and brooding adults, direct chilling may not be an imminent threat. However, wetting may exacerbate cool conditions. Thus, it is possible that the use of woody cover helped aid in shrouding water from nocturnal precipitation events and dew.

We documented neonate broods used greater amounts of woody cover and tall vegetation at roost sites, as evidenced by a positive probability of use for visual obscurity and woody cover. However, the effect of visual obscurity, but not woody cover, was reversed for juveniles observed in this study. Avian species have been documented to prefer cover that enhances crypsis over escape behavior when mobility is limited but choose sparse cover when flight and mobility are advanced (Colwell et al., 2007; Lind et al., 2010; Tirpak et al., 2005; Whittingham & Evans, 2004; Wiebe & Martin, 1998). We hypothesize the use of greater visual obscurity by neonates, and less by juveniles, could be a behavioral adaptation of predator evasion at early ages. In a similar vein, we found use of woody cover to be more important early in the nesting season when postburn recover time was short, but the interaction of age and woody cover insignificant. We believe these findings underscore the importance of woody cover in lieu of herbaceous cover when recovery time is short. The mean bareness index we observed at roost sites was 16.8%, which largely supported the findings of Carver et al. (2001) who estimated 16.7% bare ground at sites used by broods, diurnally.

Hiller and Guthery (2005) argued roosts were primarily chosen for thermoregulation and secondarily for predation. Palmer et al. (2021) mostly concurred with this notion, finding microclimates of adult bobwhite in south Texas were warmer than random locations during morning and evening hours, but substantially cooler during the mid‐day period. Counter to our predictions, our results suggested broods used roost sites that were comparatively cooler than available locations. Though we did not record ground surface temperatures during diurnal periods, our study was conducted in a relatively temperate environment not experiencing the extreme heat observed in semi‐arid regions (Figures 1 and 9). Ambient temperature collected from an on‐site weather station suggests thermal conditions do not frequently exceed various estimates of upper and lower temperature thresholds for bobwhite thermoneutrality (Figure 9; Olsen et al., 2018). The effect of temperature on resource use by bobwhite is likely more important in arid regions. Collectively, our results suggest that researchers studying ground‐dwelling precocial birds should consider the effects of life‐history characteristics and diel activity on resource use.

## 5 CONSERVATION AND MANAGEMENT PROPOSITIONS

Our results demonstrate tangible land management recommendations for conservation of bobwhite. Bobwhite broods in our study and elsewhere (Cohen et al., 2020; Sinnott et al., 2021) make short daily movements (<150 m). We found that probability of use declined with decreasing proportion of burned upland but we underscore the context of spatial scale of our resource use analysis (3.46 ha). Wellendorf and Palmer (2009) documented negative effects on adult bobwhite demographics with relatively small increases in burn scale (2.5–8 ha); effects on broods would presumably be accentuated. Moreover, Wellendorf and Palmer (2009) noted that fire applied on public lands often range from 100's to 1,000's ha in size. Thus, practitioners applying prescribed fire should consider the scale at burning occurs and species requirements.

The timing of prescribed fire may also have effects on vegetation structure. We found increased use of roost sites with greater amounts of shrub cover. Therefore, consistently hot warm season fires or other management practices that discourage the growth of shrub cover may have negative effects on bobwhite broods. This conclusion is supported by Sinnott et al. (2021) who detected lower bobwhite survival for broods that chose habitats with less shrub cover. Again, scale is important to note; these measurements were collected at the point scale (1 m^2^). Excessive shrub cover can inhibit growth of plants desirable to bobwhite such as forbs (Figure 3).

The effects of supplemental feed on brood survival remain unclear; however, we documented unequivocal use of areas close to feedlines. More research is warranted to document use of supplemental feed by broods and effects on fitness. Fallow fields appeared to have marginal effects on resource use and the effects of fallow fields on brood fitness remain unclear. Thus, we recommend land practitioners implement fallow field management on a site‐specific basis, with greater implementation in lower quality soils as suggested by Palmer et al. (2012).

The effect of mean temperature on use of nocturnal roost sites did not appear to be largely influential in our study; however, we did document nocturnal microclimate variability was associated with vegetation composition. Though we did not evaluate diurnal space use as a function of temperature, studies have unequivocally documented that diurnal space use of bobwhite in arid regions is driven by temperature, and similarly, find little effect of temperature on nocturnal space use (Guthery et al., 2005; Hiller & Guthery, 2005; Olsen et al., 2018; Palmer et al., 2021; Tanner et al., 2017). These results underscore the importance of proper habitat management that promotes both diverse floristics and vegetation physiognomy to provide accommodating microclimates for bobwhite across a wide range of life‐history stages and geographic regions.

## CONFLICT OF INTEREST

No conflicts of interest.

## AUTHOR CONTRIBUTIONS

**Bradley W. Kubecka:** Conceptualization (lead); Data curation (lead); Formal analysis (lead); Funding acquisition (lead); Investigation (lead); Methodology (lead); Visualization (lead); Writing‐original draft (lead); Writing‐review & editing (lead). **James A. Martin:** Conceptualization (supporting); Formal analysis (supporting); Investigation (supporting); Methodology (supporting); Project administration (supporting); Supervision (supporting); Visualization (supporting); Writing‐review & editing (supporting). **Theron M. Terhune:** Conceptualization (supporting); Funding acquisition (supporting); Methodology (supporting); Project administration (supporting); Supervision (supporting); Validation (supporting); Visualization (supporting); Writing‐review & editing (supporting).

## Data Availability

Data were deposited in Dyrad, https://doi.org/10.5061/dryad.vt4b8gtrn.

## References

[ece38161-bib-0001] Anderson, C. W. , Whiting, R. M. Jr , Dietz, D. R. , & Capps, R. M. (2009). Invertebrate biomass and richness in various food plot types in east Texas. National Quail Symposium Proceedings, 6, 66–77.

[ece38161-bib-0002] Anthony, C. R. , Hagen, C. A. , Dugger, K. M. , & Elmore, R. D. (2020). The effects of fire on the thermal environment of sagebrush communities. Journal of Thermal Biology, 89, 102488. 10.1016/j.jtherbio.2019.102488 32364967

[ece38161-bib-0101] Baldini, J. T. , Roberts, R. E. , & Kirkpatrick, C. M. (1952). Studies of the reproductive cycle of the bobwhite quail. Journal of Wildlife Management, 16, 91–93.

[ece38161-bib-0003] Blem, C. R. , & Zara, J. (1980). The energetics of young bobwhite (*Colinus virginianus*). Comparative Biochemistry and Physiology Part A: Physiology, 67, 611–615. 10.1016/0300-9629(80)90249-2

[ece38161-bib-0004] Booth, D. T. , Cox, S. E. , & Berryman, R. D. (2006). Point sampling digital imagery with ‘SamplePoint’. Environmental Monitoring and Assessment, 123, 97–108. 10.1007/s10661-005-9164-7 17109183

[ece38161-bib-0005] Borchelt, P. , & Ringer, R. (1973). Temperature regulation development in bobwhite quail (*Colinus virginianus*). Poultry Science, 52, 793–798. 10.3382/ps.0520793 4709423

[ece38161-bib-0006] Boyce, M. S. (2006). Scale for resource selection functions. Diversity and Distributions, 12, 269–276. 10.1111/j.1366-9516.2006.00243.x

[ece38161-bib-0007] Brennan, L. , Engstrom, R. , Palmer, W. , Hermann, S. , Hurst, G. , Burger, L. , & Hardy, C. (1998). Whither wildlife without fire? Transactions of the North American Wildlife and Natural Resources Conference, 63, 402–414.

[ece38161-bib-0008] Brooks, J. L. , & Rollins, D. (2007). Gang‐brooding in northern bobwhites in West Texas. Wilson Journal of Ornithology, 119, 137–138. 10.1676/06-040.1

[ece38161-bib-0009] Burke, J. D. , Chamberlain, M. J. , & Geaghan, J. P. (2008). Effects of understory vegetation management on brood habitat for northern bobwhites. The Journal of Wildlife Management, 72, 1361–1368. 10.2193/2007-290

[ece38161-bib-0010] Carroll, J. M. , Davis, C. A. , Elmore, R. D. , Fuhlendorf, S. D. , & Thacker, E. T. (2015). Thermal patterns constrain diurnal behavior of a ground‐dwelling bird. Ecosphere, 6, 1–15. 10.1890/ES15-00163.1

[ece38161-bib-0011] Carroll, J. M. , Davis, C. A. , Fuhlendorf, S. D. , & Elmore, R. D. (2016). Landscape pattern is critical for the moderation of thermal extremes. Ecosphere, 7, e01403. 10.1002/ecs2.1403

[ece38161-bib-0012] Carroll, J. M. , Hovick, T. J. , Davis, C. A. , Elmore, R. D. , & Fuhlendorf, S. D. (2017). Reproductive plasticity and landscape heterogeneity benefit a ground‐nesting bird in a fire‐prone ecosystem. Ecological Applications, 27, 2234–2244. 10.1002/eap.1604 28736847

[ece38161-bib-0013] Carroll, R. , Davis, C. A. , Fuhlendorf, S. D. , Elmore, R. D. , DuRant, S. E. , & Carroll, J. M. (2018). Avian parental behavior and nest success influenced by temperature fluctuations. Journal of Thermal Biology, 74, 140–148. 10.1016/j.jtherbio.2018.03.020 29801620

[ece38161-bib-0014] Carver, A. V. , Burger, L. W. Jr , & Brennan, L. A. (1999). Passive integrated transponders and patagial tag markers for northern bobwhite chicks. Journal of Wildlife Management, 63, 162–166. 10.2307/3802497

[ece38161-bib-0015] Carver, A. V. , Burger, L. W. Jr , Palmer, W. E. , & Brennan, L. A. (2001). Vegetation characteristics in seasonal‐disked fields and at bobwhite brood locations. Proceedings of the Annual Conference Southeastern Association of Fish and Wild Agencies, 28, 436–444.

[ece38161-bib-0016] Cohen, B. S. , Marquardt, D. D. , Bakner, N. W. , Perez, R. M. , & Collier, B. A. (2020). Daily Movements, Space Use, and Habitat Selection of GPS‐tagged Northern Bobwhite in Texas. Wildlife Society Bulletin, 44, 790–797. 10.1002/wsb.1137

[ece38161-bib-0017] Collins, B. M. , Williams, C. K. , & Castelli, P. M. (2009). Reproduction and microhabitat selection in a sharply declining northern bobwhite population. The Wilson Journal of Ornithology, 121, 688–695. 10.1676/09-040.1

[ece38161-bib-0018] Colwell, M. A. , Hurley, S. J. , Hall, J. N. , & Dinsmore, S. J. (2007). Age‐related survival and behavior of Snowy Plover chicks. The Condor, 109, 638–647. 10.1093/condor/109.3.638

[ece38161-bib-0019] Cornell Lab of Ornithology (2020). All about birds: Northern bobwhite overview. https://www.allaboutbirds.org/guide/Northern_Bobwhite/overview

[ece38161-bib-0020] Crawford, R. L. , & Brueckheimer, W. R. (2012). The legacy of a Red Hills Hunting Plantation: Tall Timbers Research Station & Land Conservancy. University Press of Florida.

[ece38161-bib-0021] Crouch, T. (2010). Brood habitat use and availability and daily and seasonal covey movements of northern bobwhites in east‐central Alabama. Thesis, Auburn University, Auburn, AL, USA.

[ece38161-bib-0022] Doerr, T. B. (1989). Effects of supplemental feeding on northern bobwhite populations in South Texas. Texas A&M University.

[ece38161-bib-0023] Doerr, T. B. , & Silvy, N. J. (2002). Effects of supplemental feeding on northern bobwhite populations in south Texas. National Quail Symposium Proceedings, 5, 233–240.

[ece38161-bib-0024] Faircloth, B. C. (2008). An integrative study of social and reproductive systems in Northern Bobwhite (*Colinus virginianus*): A non‐migratory, avian species bearing precocial young. University of Georgia Athens.

[ece38161-bib-0025] Faircloth, B. C. , Palmer, W. E. , & Carroll, J. P. (2005). Post‐hatching brood amalgamation in Northern Bobwhites. Journal of Field Ornithology, 76, 175–182. 10.1648/0273-8570-76.2.175

[ece38161-bib-0026] Guthery, F. S. (2002). The technology of bobwhite management: The theory behind the practice. Iowa State Press.

[ece38161-bib-0027] Guthery, F. , & Brennan, L. (2007). The science of quail management and the management of quail science. In L. Brennan (Ed.), Texas quails: Ecology and management (pp. 407–420). Texas A & M University Press.

[ece38161-bib-0028] Guthery, F. S. , Rybak, A. R. , Fuhlendorf, S. D. , Hiller, T. L. , Smith, S. G. , Puckett, W. H., Jr. , & Baker, R. A. (2005). Aspects of the thermal ecology of bobwhites in north Texas. Wildlife Monographs, 159, 1–36.

[ece38161-bib-0029] Hammond, A. D. (2001). Ecology and management of northern bobwhite broods in a longleaf pine‐wiregrass ecosystem. Mississippi State University, Department of Wildlife and Fisheries.

[ece38161-bib-0030] Hernández, F. (2020). Ecological discord and the importance of scale in scientific inquiry. Journal of Wildlife Management, 84, 1427–1434. 10.1002/jwmg.21942

[ece38161-bib-0031] Hiller, T. L. , & Guthery, F. S. (2005). Microclimate versus predation risk in roost and covert selection by bobwhites. Journal of Wildlife Management, 69, 140–149.

[ece38161-bib-0032] Hobbs, N. T. (2003). Challenges and opportunities in integrating ecological knowledge across scales. Forest Ecology and Management, 181, 223–238. 10.1016/S0378-1127(03)00135-X

[ece38161-bib-0033] Hovick, T. J. , Elmore, R. D. , Allred, B. W. , Fuhlendorf, S. D. , & Dahlgren, D. K. (2014). Landscapes as a moderator of thermal extremes: A case study from an imperiled grouse. Ecosphere, 5, 1–12. 10.1890/ES13-00340.1

[ece38161-bib-0034] Hurst, G. A. (1972). Insects and bobwhite quail brood habitat management. National Quail Symposium Proceedings, 1, 65–82.

[ece38161-bib-0035] Johnson, D. H. (1980). The comparison of usage and availability measurements for evaluating resource preference. Ecology, 61, 65–71. 10.2307/1937156

[ece38161-bib-0036] Kauffman, M. , Courtemanch, A. , & Rutledge, A. (2009). Resource selection and group association of translocated bighorn sheep in north‐central Wyoming: Does source herd matter. Wyoming Cooperative Fish and Wildlife Research Unit, University of Wyoming, Laramie, USA.

[ece38161-bib-0037] Kellner, K. (2017). jagsUI: A wrapper around ‘rjags’ to streamline JAGS analyses. https://github.com/kenkellner/jagsUI

[ece38161-bib-0038] Kennedy, M. , & Gray, R. D. (1993). Can ecological theory predict the distribution of foraging animals? A critical analysis of experiments on the ideal free distribution. Oikos, 68, 158–166. 10.2307/3545322

[ece38161-bib-0039] Kenward, R. E. (2001). Historical and practical perspectives. In J. Millspaugh & J. Marzluff (Eds.), Radio tracking and animal populations (pp. 3–12). Elsevier.

[ece38161-bib-0040] Klimstra, W. D. , & Ziccardi, V. C. (1963). Night‐roosting habitat of bobwhites. Journal of Wildlife Management, 27, 202–214. 10.2307/3798399

[ece38161-bib-0041] Kline, H. N. , Fulbright, T. E. , Grahmann, E. D. , Hernández, F. , Wester, D. B. , Brennan, L. A. , & Hehman, M. W. (2019). Temperature influences resource use by chestnut‐bellied scaled quail. Ecosphere, 10, 1–16. 10.1002/ecs2.2599

[ece38161-bib-0042] Lind, J. , Jakobsson, S. , & Kullberg, C. (2010). Impaired predator evasion in the life history of birds: Behavioral and physiological adaptations to reduced flight ability. Current Ornithology, 17, 1–30.

[ece38161-bib-0043] Lunsford, K. D. , Howell, P. E. , Roberts, T. B. , Terhune, T. M. , & Martin, J. A. (2019). Survival and growth of northern bobwhite offspring post‐translocation. Journal of Wildlife Management, 83, 1326–1335. 10.1002/jwmg.21723

[ece38161-bib-0044] Manley, S. W. , Fuller, R. S. , Lee, J. M. , & Brennan, L. A. (1994). Arthropod response to strip disking in old fields managed for northern bobwhites. Proceedings of the Annual Conference of the Southeastern Association of Fish and Wildlife Agencies, 48, 227–235.

[ece38161-bib-0045] Martin, N. , Martin, J. A. , & Carroll, J. P. (2009). Northern bobwhite brood habitat selection in South Florida.

[ece38161-bib-0046] McConnell, M. D. , Monroe, A. P. , Chandler, R. , Palmer, W. E. , Wellendorf, S. D. , Burger, L. W., Jr. , & Martin, J. A. (2018). Factors influencing Northern Bobwhite recruitment, with implications for population growth. The Auk, 135, 1087–1099. 10.1642/AUK-18-49.1

[ece38161-bib-0047] McGrath, D. J. , Terhune, T. M. , & Martin, J. A. (2017). Northern bobwhite habitat use in a food subsidized pyric landscape. Journal of Wildlife Management, 81, 919–927. 10.1002/jwmg.21254

[ece38161-bib-0048] Means, D. B. (2017). Diamonds in the rough: Natural history of the Eastern Diamondback Rattlesnake. Tall Timbers Press.

[ece38161-bib-0049] Natural Resources Conservation Service [NRCS] , U.S. Department of Agriculture (2021). Web Soil Survey. websoilsurvey.nrcs.usda.gov

[ece38161-bib-0050] Northrup, J. M. , Hooten, M. B. , Anderson, C. R. Jr , & Wittemyer, G. (2013). Practical guidance on characterizing availability in resource selection functions under a use–availability design. Ecology, 94, 1456–1463. 10.1890/12-1688.1 23951705

[ece38161-bib-0051] Olsen, B. R. , Fulbright, T. E. , Hernández, F. , Grahmann, E. D. , Wester, D. B. , & Hehman, M. W. (2018). Ground surface vs. black globe temperature in northern bobwhite resource selection. Ecosphere, 9, e02441. 10.1002/ecs2.2441

[ece38161-bib-0052] Palmer, B. J. , Fulbright, T. E. , Grahmann, E. D. , Hernandez, F. , Hehman, M. W. , & Wester, D. B. (2021). Vegetation structural attributes providing thermal refugia for northern bobwhites. The Journal of Wildlife Management, 85, 543–555. 10.1002/jwmg.22006

[ece38161-bib-0053] Palmer, W. E. , Carroll, J. P. , Sisson, D. C. , Wellendorf, S. D. , Terhune, T. M. , Ellis‐Felege, S. N. , & Martin, J. A. (2019). Reduction in meso‐mammal nest predators improves northern bobwhite demographics. Journal of Wildlife Management, 83, 646–656. 10.1002/jwmg.21627

[ece38161-bib-0054] Palmer, W. , & Sisson, D. C. (2017). Tall Timbers' Bobwhite quail management handbook. Tall Timbers Press.

[ece38161-bib-0055] Palmer, W. E. , Sisson, D. C. , Wellendorf, S. D. , Bostick, A. M., III , Terhune, T. M. , & Crouch, T. L. (2012). Habitat selection by northern bobwhite broods in pine savanna ecosystems.

[ece38161-bib-0056] Parsons, D. S. , Whiting, R. M., Jr. , Liu, X. , & Dietz, D. R. (2000). Food plot use by juvenile northern bobwhites in east Texas. National Quail Symposium Proceedings, 4, 71–74.

[ece38161-bib-0057] Perkins, R. , Boal, C. , Rollins, D. , & Perez, R. M. (2014). Northern bobwhite predator avoidance behavior in response to varying types of threat. The Journal of Wildlife Management, 78, 1272–1281. 10.1002/jwmg.766

[ece38161-bib-0100] Petrides, G. A. , & Nestler, R. B. (1943). Age determination in juvenal bobwhite quail. American Midland Naturalist, 30, 774–782.

[ece38161-bib-0058] PRISM Climate Group, Oregon State University (2020). Time series values for individual locations. http://www.prism.oregonstate.edu/explorer/

[ece38161-bib-0059] R Core Team (2020). R: A language and environment for statistical computing. R Foundation for Statistical Computing. http://www.r‐project.org/index.html

[ece38161-bib-0060] Rettie, W. J. , & Messier, F. (2000). Hierarchical habitat selection by woodland caribou: Its relationship to limiting factors. Ecography, 23, 466–478. 10.1111/j.1600-0587.2000.tb00303.x

[ece38161-bib-0061] Roseberry, J. L. , & Klimstra, W. D. (1984). Population ecology of the bobwhite. Southern Illinois University Press.

[ece38161-bib-0062] Rother, M. T. , Huffman, J. M. , Guiterman, C. H. , Robertson, K. M. , & Jones, N. (2020). A history of recurrent, low‐severity fire without fire exclusion in southeastern pine savannas, USA. Forest Ecology and Management, 475, 118406. 10.1016/j.foreco.2020.118406

[ece38161-bib-0063] Sandercock, B. K. , Jensen, W. E. , Williams, C. K. , & Applegate, R. D. (2008). Demographic sensitivity of population change in northern bobwhite. Journal of Wildlife Management, 72, 970–982. 10.2193/2007-124

[ece38161-bib-0064] Sands, J. P. , Brennan, L. A. , Hernández, F. , Kuvlesky, W. P. Jr , Gallagher, J. F. , & Ruthven, D. C., III (2012). Impacts of introduced grasses on breeding season habitat use by northern bobwhite in the South Texas plains. Journal of Wildlife Management, 76, 608–618. 10.1002/jwmg.305

[ece38161-bib-0065] Sauer, J. R. , Pardieck, K. L. , Ziolkowski, D. J. Jr , Smith, A. C. , Hudson, M.‐A.‐R. , Rodriguez, V. , Berlanga, H. , Niven, D. K. , & Link, W. A. (2017). The first 50 years of the North American Breeding Bird Survey. The Condor, 119, 576–593. 10.1650/CONDOR-17-83.1

[ece38161-bib-0066] Scott, T. G. (1985). Bobwhite thesaurus. International Quail Foundation.

[ece38161-bib-0067] Singh, A. , Hines, T. C. , Hostetler, J. A. , Percival, H. F. , & Oli, M. K. (2011). Patterns of space and habitat use by northern bobwhites in South Florida, USA. European Journal of Wildlife Research, 57, 15–26. 10.1007/s10344-010-0393-x

[ece38161-bib-0068] Sinnott, E. A. , Weegman, M. D. , Thompson, T. R. , & Thompson, F. R. (2021). Resource selection and movement by northern bobwhite broods varies with age and explains survival. Oecologia, 195(4), 937–948. 10.1007/s00442-021-04893-z 33677683

[ece38161-bib-0069] Sisson, D. C. , Stribling, H. L. , & Speake, D. W. (2000). Effects of supplemental feeding on home range size and survival of northern bobwhites in south Georgia. National Quail Symposium Proceedings, 4, 128–131.

[ece38161-bib-0070] Smith, M. D. , Hammond, A. D. , Burger, L. , Palmer, W. E. , Carver, A. V. , & Wellendorf, S. D. (2003). A technique for capturing northern bobwhite chicks. Wildlife Society Bulletin, 31, 1054–1060.

[ece38161-bib-0071] Spiers, D. E. , Adams, T. , & Ringer, R. K. (1985). Homeothermic development in the bobwhite (*Colinus virginianus*). Journal of Comparative Biochemistry Physiology Part A: Physiology, 81, 921–927. 10.1016/0300-9629(85)90931-4

[ece38161-bib-0072] Stoddard, H. L. (1931). The bobwhite quail: Its habits, preservation and increase. Charles Scribner and Sons.

[ece38161-bib-0073] Strickland, M. D. , & McDonald, L. L. (2006). Introduction to the special section on resource selection. The Journal of Wildlife Management, 70, 321–323.

[ece38161-bib-0074] Tanner, E. P. , Elmore, R. D. , Fuhlendorf, S. D. , Davis, C. A. , Dahlgren, D. K. , & Orange, J. P. (2017). Extreme climatic events constrain space use and survival of a ground‐nesting bird. Global Change Biology, 23, 1832–1846. 10.1111/gcb.13505 27633847

[ece38161-bib-0075] Taylor, J. D., II , & Burger, L. W., Jr. (2000). Habitat use by breeding northern bobwhites in managed old‐field habitats in Mississippi. National Quail Symposium Proceedings, 4, 7–15.

[ece38161-bib-0076] Taylor, J. S. , Church, K. E. , & Rusch, D. H. (1999). Microhabitat selection by nesting and brood‐rearing northern bobwhite in Kansas. Journal of Wildlife Management, 63, 686–694. 10.2307/3802658

[ece38161-bib-0077] Terhune, T. M. , Caudill, D. , Terhune, H. V. , & Martin, J. A. (2020). A modified suture technique for attaching radiotransmitters to Northern Bobwhite Chicks. Wildlife Society Bulletin, 44, 396–405. 10.1002/wsb.1077

[ece38161-bib-0078] Terhune, T. M. , Chandler, R. B. , & Martin, J. A. (2017). Estimates of Northern Bobwhite neonate survival. National Quail Symposium Proceedings, 8, 60.

[ece38161-bib-0079] Terhune, T. M. , Palmer, W. E. , & Wellendorf, S. D. (2019). Northern Bobwhite Chick survival and effects of weather. Journal of Wildlife Management, 83, 963–974. 10.1002/jwmg.21655

[ece38161-bib-0080] Tirpak, J. M. , Giuliano, W. M. , & Miller, C. A. (2005). Nocturnal roost habitat selection by ruffed grouse broods. Journal of Field Ornithology, 76, 168–175. 10.1648/0273-8570-76.2.168

[ece38161-bib-0081] Tsalyuk, M. , Kilian, W. , Reineking, B. , & Getz, W. M. (2019). Temporal variation in resource selection of African elephants follows long‐term variability in resource availability. Ecological Monographs, 89, e01348. 10.1002/ecm.1348

[ece38161-bib-0082] Van der Merwe, J. , & Marshal, J. P. (2012). Hierarchical resource selection by impala in a savanna environment. Austral Ecology, 37, 401–412. 10.1111/j.1442-9993.2011.02297.x

[ece38161-bib-0083] Wellendorf, S. D. , & Palmer, W. E. (2009). Effects of two burn scales on northern bobwhite demographic parameters and home range size. National Quail Symposium Proceedings, 6, 271–281.

[ece38161-bib-0084] Wellendorf, S. D. , Palmer, W. E. , & Bostick, A. M. III (2017). Effects of supplemental feeding on breeding season home ranges and resource selection of northern bobwhites. National Quail Symposium Proceedings, 8, 187–195.

[ece38161-bib-0085] Wellendorf, S. D. , Palmer, W. E. , & Sisson, D. C. (2017). Supplemental feeding. In W. E. Palmer & D. C. Sisson (Eds.), Tall Timbers’ Bobwhite quail management handbook. Tall Timbers Press.

[ece38161-bib-0086] West, A. S. , Keyser, P. D. , & Morgan, J. J. (2012). Northern bobwhite survival, nest success, and habitat use in Kentucky during the breeding season. National Quail Symposium Proceedings, 7, 217–222.

[ece38161-bib-0087] White, G. , & Garrott, R. (1990). Analysis of wildlife radio‐tracking data. Academic Press.

[ece38161-bib-0088] Whittingham, M. J. , & Evans, K. L. (2004). The effects of habitat structure on predation risk of birds in agricultural landscapes. Ibis, 146, 210–220. 10.1111/j.1474-919X.2004.00370.x

[ece38161-bib-0089] Wiebe, K. L. , & Martin, K. (1998). Costs and benefits of nest cover for ptarmigan: Changes within and between years. Animal Behaviour, 56, 1137–1144. 10.1006/anbe.1998.0862 9819329

[ece38161-bib-0090] Williams, C. K. , Lutz, R. S. , Applegate, R. D. , & Rusch, D. H. (2000). Habitat use and survival of northern bobwhite (*Colinus virginianus*) in cropland and rangeland ecosystems during the hunting season. Canadian Journal of Zoology, 78, 1562–1566.

[ece38161-bib-0091] Yeiser, J. M. , Jackson, A. L. , Sisson, D. C. , Terhune, T. M. , & Martin, J. A. (2020). Predation management and spatial structure moderate extirpation risk and harvest of Northern Bobwhite. The Journal of Wildlife Management, 85, 50–62. 10.1002/jwmg.21964

